# 1^st^ International Symposium on Stress-Associated RNA Granules in Human Disease and Viral Infection

**DOI:** 10.3390/v6093500

**Published:** 2014-09-23

**Authors:** Bruce W. Banfield, Andrew J. Mouland, Craig McCormick

**Affiliations:** 1 Department of Biomedical and Molecular Sciences, Queen’s University, Kingston, ON K7L 3N6, Canada; 2 HIV-1 RNA Trafficking Laboratory, Lady Davis Institute at the Jewish General Hospital, Montréal, QC H3T 1E2, Canada; 3 Department of Medicine, McGill University, Montréal, QC H3A 0G4, Canada; 4 Department of Microbiology and Immunology, McGill University, Montréal, QC H3A 0G4, Canada; 5 Department of Microbiology and Immunology, Dalhousie University, 5850 College Street, Halifax, NS B3H 4R2, Canada; 6 Beatrice Hunter Cancer Research Institute, Dalhousie University, 5850 College Street, Halifax, NS B3H 4R2, Canada

**Keywords:** stress granules, p-bodies, viruses, cancer, neurological disorders

## Abstract

In recent years, important linkages have been made between RNA granules and human disease processes. On June 8-10 of this year, we hosted a new symposium, dubbed the 1^st^ International Symposium on Stress-Associated RNA Granules in Human Disease and Viral Infection. This symposium brought together experts from diverse research disciplines ranging from cancer and neuroscience to infectious disease. This report summarizes speaker presentations and highlights current challenges in the field.

## 1. Introduction

**Figure 1:**
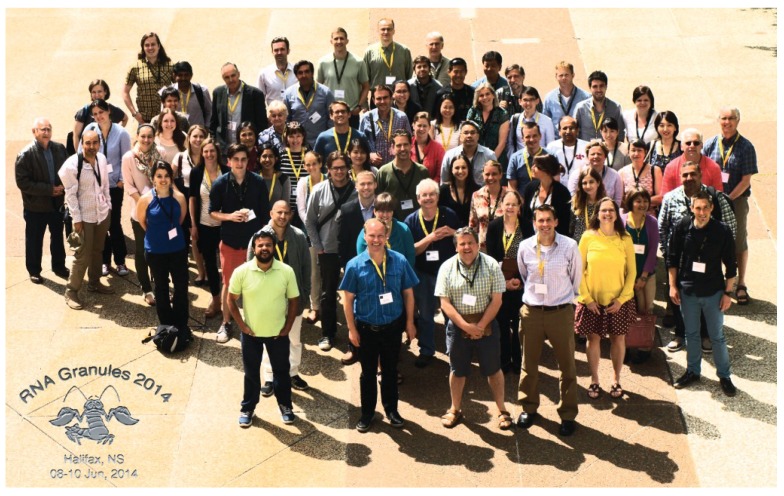
*RNA Granules 2014* group photo.

The field of RNA granule biology and how it impacts human disease is developing at a rapid pace. RNA granules are dynamic, membrane-free cytoplasmic structures that play important roles in controlling the timing and location of mRNA translation. Like the granules that they study, a diverse and energetic group of scientists paused their experiments and congregated (aggregated?) in Halifax, Nova Scotia this past June to participate in the 1^st^ International Symposium on Stress-Associated RNA Granules in Human Disease and Viral Infection, also known as *RNA Granules 2014* (Figure 1, group photo). The goal of this meeting was to provide a comprehensive update of recent insights into the molecular mechanisms of RNA granule assembly/disassembly and function as they relate to human health. The meeting brought together those who study RNA granule dynamics in neurological disorders, cancer and infectious diseases, and introduced them to long-time leaders in the field including Richard (Rick) Lloyd, Paul Anderson and Nancy Kedersha (note: readers are encouraged to consult authoritative reviews by these individuals [1,2]). A spirit of open communication prevailed at the symposium, along with recognition that researchers operating in different disciplines had much to learn from each other.

## 2. Crosstalk between RNA Granules and Innate Immunity

**Figure 2:**
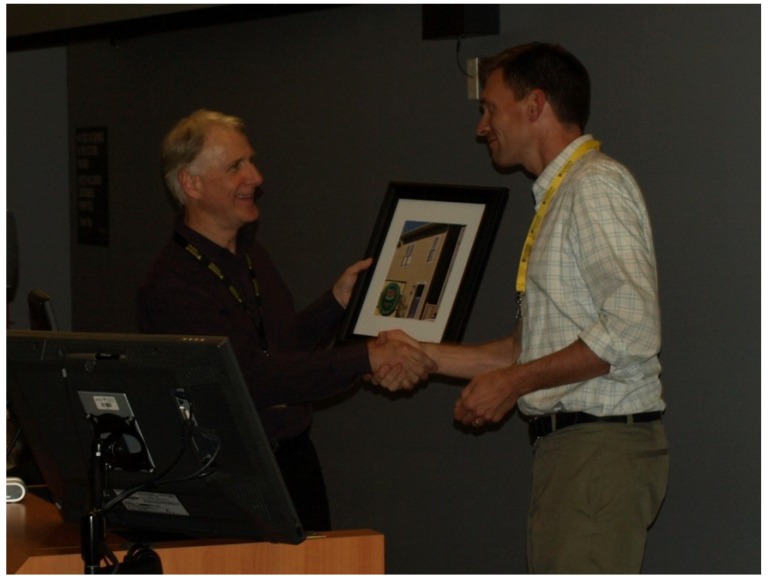
Dr. Richard (Rick) Lloyd (**left**) receives gift following his Keynote Address, a framed photo of the registered heritage property at 2380 Moran Street in Halifax, Nova Scotia. Built in 1866, it was the childhood home of pioneering scientist Oswald Avery, who discovered that genetic material is comprised of nucleic acid, not protein as was previously thought.

The symposium opened with a Keynote Lecture from Rick Lloyd from Baylor College of Medicine, well known for his discovery of the mechanism whereby picornaviruses (such as poliovirus) prevent stress granule (SG) formation by cleavage of essential SG-constituent proteins like GTPase Activating Protein (SH3 Domain) Binding Protein 1 (G3BP1) [3]. Rick reviewed the origins of the field and provided the diverse audience with a useful review of the properties of SGs and another commonly studied RNA granule known as the processing body (p-body, or PB), and the accepted standards in the field for discrimination of these structures. With infectious enthusiasm for the topic, Rick clearly articulated the outstanding questions in the field with regard to cellular stress responses and regulation of RNA granule assembly and disassembly. With the table nicely set, Rick dug into the most intriguing and controversial aspect of SG biology, namely its role in regulating innate immune responses to viral infection. A growing number of viral gene products have been shown to block SG formation, and disabling these gene products strongly correlates with arrested viral gene expression and a failure to efficiently replicate. In these situations, certain viral proteins and nucleic acids, along with host factors involved in pathogen sensing, such as the dsRNA-binding protein kinase R (PKR) and the RNA helicases RIG-I and MDA5, can be sequestered in SGs. Rick carefully weighed the evidence for the new hypothesis that SGs are capable of signaling innate immune responses as part of a primordial integrated stress-immune activation response. In this context, he described ongoing work in his lab on G3BP1, which is emerging as a new antiviral protein which functions against multiple RNA viruses. It appears that a feature of G3BP1 antiviral activity is activation of two arms of the innate immune response through PKR and NF-kB. G3BP1 is normally recruited to nascent SGs immediately following phosphorylation of eIF2α by one of four eIF2α kinases (PKR, PERK, HRI, GCN2) and arrest of translation initiation, but Rick reported that ectopic expression of G3BP1 creates a feed-forward loop that enhances eIF2α phosphorylation and drives the formation of larger SGs with more potent antiviral activities [4]. The precise mechanism of G3BP1-mediated eIF2α phosphorylation remains an active area of investigation. Rick also presented recent mechanistic insights into G3BP1-mediated NFkB activation, which involves binding IkB via its acidic domain and promotion of NFkB nuclear translocation and activation of interferon-stimulated genes (ISGs). He also discussed the evidence for regulation of G3BP1 activities by post-translational modifications, such as methylation and phosphorylation. This overview of the major unanswered questions and emerging ideas in the field provided a great start to the symposium, and spawned vigorous discussions that continued on through the night. Before being granted his freedom to enjoy wine and cheese, Rick was presented with a gift of a framed photo of a Halifax heritage property, a 19th century row-house that was the childhood home of Oswald Avery (Figure 2).

## 3. Fundamental Regulation of Stress Granule Assembly and Disassembly

There could be no better individual to lead the first session on biogenesis and dynamics of RNA granules than Paul Anderson from Brigham and Women’s Hospital, whose laboratory first put RNA granules on the map, and spurred the imagination of many investigators around the world. Paul focused on the cytoprotective effects of SGs, which have been largely attributed to reprogramming of gene expression and sequestration of pro-apoptotic proteins. He discussed the evidence that cytoprotective SGs play roles in cancer cell resistance to chemotherapy and radiation. Screening of chemotherapeutic drugs revealed that several of them perturbed SG formation by distinct mechanisms. One of the drugs, vinorelbine, disrupts microtubules and, hence, cancer cell proliferation, but also induces SG assembly in an eIF2α-dependent fashion, thereby eliciting unhelpful cytoprotective effects. Arsenic (As) triggers oxidative stress and HRI-dependent SG formation, while the adjacent element on the periodic table, selenium (Se), remains largely unexplored to date. Notably, Se has recently been implicated as a cancer chemoprotective factor [5]. By investigating the properties of Se, Paul discovered that Se-induced SGs have a different composition than classical As-induced SGs [6]. For example, Se-induced SGs contain G3BP1, but not eIF3b. At the same time, Se negatively regulated mTOR activity by various indices (*i.e.*, depressed phosphorylation of S6K and 4EBP). Interestingly, the mTORC1/2 inhibitor Torin1 inhibited SG formation; combined treatment of cancer cells with Torin1 and vinorelbine inhibited SG assembly while maintaining a translation blockade. These promising early studies bolster the idea that SGs are central players in cancer chemotherapy and radiotherapy resistance, which could be effectively manipulated by combinations of SG-modulating drugs.

RNA granule assembly and disassembly is tightly regulated but for the most part the molecular mechanisms remain to be elucidated. With an encyclopedic knowledge of the properties of RNA granules, Paul Anderson’s longtime colleague Nancy Kedersha at Brigham and Women’s Hospital offered new insights into the dynamic assembly/disassembly of SGs through her study of the SG nucleating protein G3BP1. Dephosphorylation of G3BP1 at Ser149 acts as a switch that permits oligomerization and SG nucleation [7]. G3BP1 also interacts with a plethora of other SG-associated proteins including Caprin1 and USP10. Nancy presented preliminary data indicating differential regulation of SG nucleation by these G3BP1 binding partners, whereby Caprin1 and USP10 bind the G3BP-NTF2-like domain in a competitive manner, with opposite results; G3BP1:Caprin1 complexes promote SG formation, whereas G3BP1:USP10 complexes inhibit SG formation. Moreover, she provided mechanistic insights into these differential effects, demonstrating that G3BP1:USP10 complexes can link 40S ribosomal subunits and PABP, which may prime stalled mRNPs for translation, which would have the effect of inhibiting SG formation.

Ultraviolet (UV) irradiation causes activation of eIF2α kinases and arrest of translation initiation [8], resulting in SG formation [9], but molecular mechanisms remain obscure. Rachid Mazroui from Université Laval presented evidence for a new class of RNA granules that form upon exposure to sublethal doses of ultraviolet C (UVC) irradiation, termed mammalian ultraviolet C granules (mUVCGs). In this system, mUVCG formation did not coincide with eIF2α phosphorylation or profound inhibition of translation. Intriguingly, mUVCG formation correlated with G1 cell cycle arrest, whereas mUVCG disassembly correlated with S-phase entry. These observations suggest that these mUVCGs could possess a unique role in the cell cycle or in other aspects of cell metabolism.

Poly(ADP-ribose) (PAR) is an RNA-like polymer of ADP ribose subunits synthesized by PAR polymerases (PARPs) using NAD+ as a substrate. Poly(ADP-ribosyl)ation (PARylation), the covalent attachment of PAR to proteins, can range from two to several hundred ADP-ribose units in length. Seventeen PARPs have been identified in the human genome, however, the specific functions of the majority of these enzymes is unclear. The most well studied PARP, PARP1, functions early in DNA damage responses. Upon recruitment to sites of damaged DNA PARP1 undergoes auto-PARylation, which coincides with PARylation of other proximal proteins. This focus of PARylated proteins serves as a platform for the recruitment of additional PAR binding proteins involved in the coordination and execution of DNA damage repair. Unlike PARP-1, the majority of PARPs localize to the cytoplasm so it is, perhaps, not surprising that other roles for PAR have emerged in recent years [10,11]. Anthony Leung from Johns Hopkins University showed us that PARylation is required for the formation of SGs [12]. Specifically, five different PARPs were found to localize to SGs, along with two PAR glycohydrolase (PARG) enzymes capable of degrading PAR polymers. Anthony demonstrated that SG formation is sensitive to the levels of these enzymes; overexpression of PARPs caused SG formation, whereas PARG overexpression inhibited SG assembly and silencing PARG expression stabilized SGs. A number of SG components were demonstrated to either be modified by PAR or to bind to PAR. Thus, Anthony proposes that PARylation initiates SG formation through local nucleation of proteins proximal to the sites of PAR modification [13]. Anthony also discussed the difficulties in identifying PARylated proteins by mass spectroscopy due to the lack of unique mass signatures associated with this modification. He presented a new unbiased approach for identifying PARylated (as well as mono-ADP-ribosylated) proteins that relies on the enzymatic product of phosphodiesterase-treated ADP-ribose that can subsequently be enriched using phosphoproteomic techniques [14]. These new procedures have great potential to reveal the complete spectrum of PARylated proteins produced in cells exposed to a variety of stimuli, including those that induce cell stress.

Consistent with Rick Lloyd’s intimation that RNA granules play roles in defenses against cell stresses and viral infections, Archa Fox from The University of Western Australia discussed her research on paraspeckles, subnuclear RNA granules that have been shown to have a role in cell defence against a variety of viruses. She described the details of the assembly of paraspeckles, nuclear RNA granules that are thought to function, at least in part, to sequester transcription factors. Archa showed that paraspeckle assembly was dependent on nuclear paraspeckle assembly transcript (NEAT1), and this long noncoding RNA is transcriptionally activated by many factors including p53 through direct binding to the promoter. Interestingly, p53 null cells continued to form paraspeckles, suggesting a complex regulation of paraspeckles that will respond to various types of cell stress including virus infections.

## 4. RNA Granules in Neurological Disorders and Cancer

Colin Crist from McGill University opened the next session by reporting on facets of skeletal muscle regeneration from muscle stem cells that rely on sequestration of critical factors in SG-like RNA granules, that include FMRP, DDX6 and TIA1 amongst other classical SG marker proteins [15]. MicroRNAs regulate the expression of the myogenic determination gene myf5 and these RNAs are sequestered in SGs until stem cells activate to regenerate muscle. Upon activation, the RNA granules disassemble to promote myf5 mRNA recruitment to the translation apparatus, allowing the muscle stem cells to rapidly enter the myogenic program. This work indicates that RNA granules play key roles in tissue homeostasis.

The Montréal RNA granule connection was reinforced by Université de Montréal’s Christine Vande Velde’s talk on the amyotrophic lateral sclerosis (ALS) disease-associated gene product, TAR DNA-binding protein of 43 kDa **(**TDP-43) [16]. ALS is a neurodegenerative disease that leads to loss of motor neurons in the motor cortex, brainstem and spinal cord, muscle atrophy, respiratory failure and death and is often associated with mutations in TDP-43 [17]. TDP-43 alterations are met with impaired aggregation and assembly of SGs. Christine provided evidence that an association exists between ALS and RNA metabolism via changes in the size and abundance of SGs. This work suggests new opportunities for therapeutic intervention in ALS by modulating the dynamic assembly and disassembly of RNA granules.

Of course, neuronal RNA granules have historically been implicated in RNA transport along cytoskeletal elements in these elongated cells to control localized mRNA translation [18]. Huntington’s disease (HD) is characterized by a CAG triplet repeat expansion within the huntingtin (HTT) gene and this increase in polyglutamine repeats is the causative factor in the pathogenesis of HD. Naoko Tanese, from New York University School of Medicine, found that the HD-associated protein, huntingtin (Htt), co-trafficked with RNA transport granules in dendrites and also co-associated with constituents of these granules [19]. A correlation between Htt in these granules provides some support for a coupling between Htt function and RNA fate in this neurodegenerative disease, as Naoko and colleagues reported earlier [20].

In the last talk of this session, Poul Sorensen from the University of British Columbia focused on Y-box binding protein 1 (YB-1), a highly conserved member of the cold shock domain-containing family of proteins. Overexpression of YB-1 in tumor samples correlates with drug resistance and poor survival, and* in vitro* studies have shown that YB-1 supports epithelial-mesenchymal transition (EMT) in breast cancer cells. YB-1 is a nucleocytoplasmic shuttling protein that binds to mRNA and affects the translation of many genes, but precise mechanisms of translation control remain elusive to date. Poul reported that YB-1 interacts with SG proteins including G3BP1 and TIA-1 as well as polyadenylated mRNA, and translocates to SGs in response to diverse stresses, including oxidative stress and hypoxia. Previous studies from the Sorensen lab showed that YB-1 binds and promotes cap-independent translation of mRNAs involved in EMT [21]. Poul presented data demonstrating that YB-1 directly binds to the 5’ UTR of G3BP1 and promotes translation, thereby controlling G3BP1 availability for SG assembly. This notion was supported by data from tumor xenografts showing strong correlation between YB-1 downregulation and inhibition of SG formation* in vivo*.

## 5. RNA Granules in Infectious Diseases

After an all-too-short poster session that featured many interesting studies ranging from ALS to hearing loss and caliciviruses to rotaviruses, the afternoon oral session began. This session featured talks focused on RNA granules in the context of infectious disease.

The Roizman laboratory demonstrated a decade ago that TIA-1/TIAR containing cytoplasmic granules form late in infection in cells infected with a herpes simplex virus type 1 (HSV-1) mutant defective in the viral ribonuclease, vhs [22]. More recently, these findings were extended by the Smiley group, who showed that these granules were *bona fide* SGs [23]. The formation of SGs in cells was accompanied by a dramatic reduction in the translation of late HSV-1 gene products during infection. Jim Smiley, from the University of Alberta, presented evidence that the failure of vhs null virus infected cells to synthesize late virus proteins is due to mRNA saturation of the translational machinery at late times post infection and that the lack of late protein translation is due to a failure of late viral transcripts to compete for the translational machinery, rather than due to specific features contained within late mRNAs [24]. The implication is that by degrading viral and cellular mRNAs, vhs facilitates the availability of translation factors that are not otherwise overwhelmed/occupied by the abundantly produced viral mRNAs synthesized during the early stages of virus infection. However, because SGs form in the absence of vhs, presumably whatever translation factors are limiting in vhs null virus infected cells would not be those required for the formation of pre-initiation complexes.

In a related story, Renée Finnen, from the Banfield lab at Queen's University, described how an infecting virion component of HSV-2 actively prevents SGs from forming in infected cells [25]. As it happens the virion component required for prevention of SG formation is the HSV-2 ortholog of the viral ribonuclease vhs. Similar to the situation in HSV-1, infection of cells with HSV-2 mutants defective in vhs results in robust formation of SGs. Characterization of these SGs revealed that they contained at least two virus encoded proteins, ICP27 and Us3. While the presence of ICP27 in these structures might have been expected because of its well-known functions as an RNA binding protein and its interactions with components of the translational apparatus, the presence of the viral serine/threonine kinase Us3 in these structures was unexpected [26]. These findings may suggest a role for Us3 in translational regulation in herpesvirus infected cells.

All herpesviruses establish persistent infection of their hosts through latency, a relatively quiescent mode of infection in which viral gene expression is limited and the genome is maintained as an episome tethered to chromatin. After two talks focused on lytic herpesvirus replication, Jennifer Corcoran from the McCormick lab at Dalhousie University switched focus to herpesvirus latency and the regulation of p-bodies. Through study of the cancer-causing herpesvirus Kaposi’s sarcoma-associated herpesvirus (KSHV), Jenn discovered that p-body dispersion is a feature of KSHV infection [27]. The hallmark of Kaposi’s sarcoma is the latently infected endothelial cell, which displays marked cytoskeletal alterations and elaboration of pro-inflammatory cytokines and angiogenic factors that support tumorigenesis. Jenn revealed that a product of the latent gene expression program supports these secretory and migratory phenotypes while simultaneously causing dispersion of p-bodies. Ongoing work will address the molecular mechanism of p-body dispersion and functional consequences for tumor initiation.

Moving away from the herpesviruses, Gerald McInerney of the Karolinska Institute, told us about a conserved amino acid motif, (L/I)TFGDFD, initially identified in the non-structural protein 3 (nsP3) of the alphaviruses Semliki Forest virus and Chikungunya virus, that facilitates binding of nsP3 to G3BP [28]. The critical features of this motif were identified and, satisfyingly, an *in silico* search for other proteins containing this motif identified other known G3BP binding cellular proteins. Other viral proteins containing this motif were experimentally verified as G3BP-binding and SG-inhibiting proteins. However, the significance of these interactions for viral replication cycles awaits further investigation.

It is well appreciated that invasive bacterial pathogens, such as *Shigella* and *Salmonella* species, induce an amino acid starvation response that triggers host defense through autophagy [29]. In a surprising twist to this tale, Stephen Girardin from the University of Toronto shared with us how amino acid starvation impacts the host cell splicing machinery in bacterially infected animal cells. The data so far suggest that this occurs through interference with SMN-mediated U snRNA maturation in the cytosol, resulting in the formation of cytosolic U bodies—RNA granules that contain uridine-rich small nuclear ribonucleoproteins that are thought to serve as sites for their assembly, modification, and/or storage.

The final two talks of the session featured studies on hepatitis C virus (HCV). The first was presented by Alessia Ruggieri of the University of Heidelberg, who elaborated on her fascinating study on the dynamic formation and dissolution of SGs that occurs in HCV infected cells exposed to type 1 interferon (IFN-α) [30]. These oscillations in SG assembly and disassembly correspond to cycles of active and silenced translation in infected cells and depend on the interferon inducible, dsRNA sensing, eIF2α kinase PKR and can be negatively regulated by the eIF2α phosphatase component GADD34. Interestingly, this oscillating stress response is a conserved host cell reaction to various single-stranded RNA virus infections, however, the oscillation frequencies vary between viruses. Using mathematical modeling, attempts are being made to identify viral and cellular parameters that influence the magnitude and frequency of virus-induced SG oscillations.

The final talk of the session was delivered by Yasuo Ariumi from Kumamoto University, who demonstrated the relocalization of SG and p-body components to the periphery of lipid droplets produced in HCV infected cells [31]. The surfaces of lipid droplets have been proposed to serve as platforms for HCV virion replication and assembly. Knockdown of several p-body and SG components resulted in inhibition of HCV RNA replication, which intriguingly suggests that some of these RNA granule components are being usurped by the virus to facilitate its replication.

**Figure 3:**
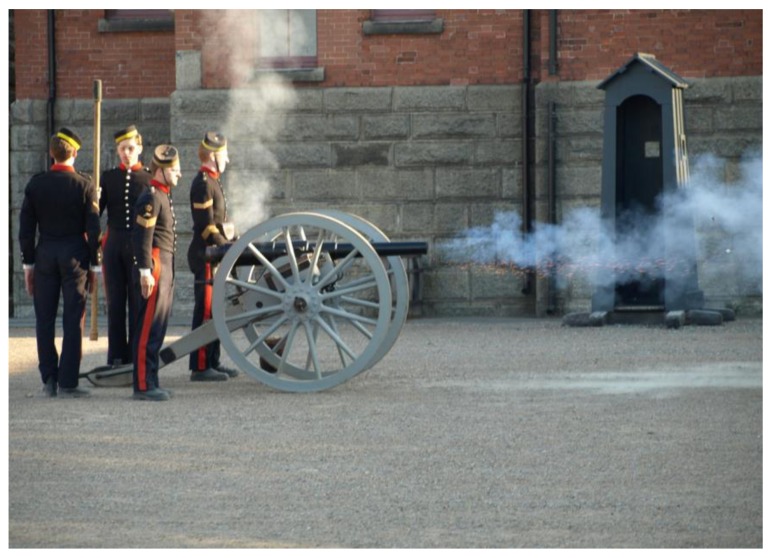
The 78^th^ Highlanders (Halifax-Citadel) demonstrate the firing of a field gun in close proximity to symposium attendees. We were all apprehensive when, after firing their first two shots, they pivoted the cannon, rushed towards us, and fired two more deafening shots. No scientists were harmed in this demonstration.

At this point we were off to the banquet in style. We gathered outside the lecture hall on what was a gorgeous afternoon where we were met by a piper (James) and drummer (Daniel) from the 78^th^ Highlanders Pipe Band, who led us through the streets of Halifax to the Halifax Citadel National Historic Site. “The Citadel”, as it is known, is a star-shaped fortress built in 1749 to counterbalance the French stronghold of Louisbourg on Ile-Royale (modern-day Cape Breton Island). As we passed through the stone gates the music became a deafening roar, and we emerged into an open courtyard. Upon our arrival, Highlanders provided historic tours of the ramparts and entertained us with cannon fire (Figure 3), which was followed by a banquet that featured local delicacies.

Day 3 of the symposium was largely focused on the dynamic interactions between RNA viruses and SGs/PBs. The day began with Margo Brinton from Georgia State University, who described how West Nile virus (WNV) inhibits SG formation. WNV infection strongly induces anti-oxidant pathways, which is sufficient to inactivate the reactive oxygen species (ROS) response induced by arsenite treatment, thereby preventing SG formation. Moreover, Margo reported that small perinuclear foci located close to WNV replication complexes form in some cell lines and contain many SG components, including TIAR, G3BP, PABP, eIF3A and caprin-1, but not HuR. Following her presentation, Margo advertised a 2016 Keystone Symposium that she is co-organizing with Drs. Beth Levine and Sandra Weller, entitled “*Cell Stress Responses and Infectious Agents*”. Many in attendance saw this as an excellent opportunity to present the next chapter of their work on RNA granules and viruses.

There is emerging evidence that RNA granule biology is central to the proper functioning of the mammalian RNA silencing machinery; many components of this machinery can be found in SGs and PBs. Tom Hobman from the University of Alberta, an expert in Rubella virus and West Nile virus, presented his ongoing fundamental studies of the regulation of RNA silencing pathways. Tom’s group was the first to report the discovery and characterization of human Argonaute-2 (hAgo2), formerly known as GERp95 [32], the catalytic core component of the RNA-induced silencing complex (RISC). hAgo2 that is engaged in RNA silencing is recruited to SGs and PBs, but the significance of this phenomenon is not entirely clear at this point. Using a combination of biochemical and genetic approaches, Tom’s laboratory identified multiple trans-acting factors that affect Ago2-dependent RNA silencing, including multiple Hsp90 complexes that regulate RNAi at different stages, as well as a plethora of evolutionarily conserved kinases and phosphatases.

Eric Jan from the University of British Columbia presented evidence that viral modulation of SGs is not limited to viruses of mammals. The insect virus Cricket paralysis virus (CrPV), a member of the dicistrovirus family, causes rapid shutoff of host translation in Drosophila S2 cells, allowing viral mRNAs to gain priority access over the translation apparatus. By monitoring fruit fly homologs of canonical SG and PB constituent proteins, Eric observed general dispersal of both SGs and PBs during dicistrovirus infection, even in response to challenge with heat shock, oxidative stress, and pateamine A treatment [33]. Many viral proteins have been reported to localize to nascent SGs, and Eric reported here that CPV 3C protease was one such protein, although the functional significance of this observation remains unclear.

Fernando Valiente-Echeverría from the Mouland lab at McGill University provided new mechanistic insight into how HIV-1 blocks SG assembly* in vitro* and* ex vivo* in patient samples. Fernando, who has taken a new position at Universidad de Chile, showed a new and completely unexpected role for the major structural protein of HIV-1, group specific antigen or Gag. He demonstrated a strong effect of Gag in inhibiting SG assembly even when SG assembly was forced by the over expression of G3BP-1 or TIAR [34]. Results derived from biochemical, virological and microscopic analyses consistently demonstrated a Gag-specific effect on the assembly and disassembly of SG that were dependent on interactions between the host factors eEF2 and G3BP-1. While the implication of the eEF2-Gag interaction is novel, the recruitment or targeting of G3BP during virus replication seems to be a recurrent theme for many viruses [3,35,36]. Revealing how HIV and other retroviruses [37] prevent SG assembly will provide important clues about fundamental regulation of SG formation, while at the same time identifying potential targets for therapeutic intervention.

Influenza A virus (IAV) polymerase complexes function in the nucleus of infected cells, generating mRNAs that bear 5’ caps and poly(A) tails, which are exported to the cytoplasm and translated by host machinery. For this reason, IAV gene expression would be predicted to be extremely limited in times of stress, and IAV mRNAs recruited to nascent SGs. Indeed, Denys Khaperskyy from the McCormick lab at Dalhousie University showed that at early stages of infection both viral and host mRNAs are sensitive to drug-induced translation arrest and SG formation [38]. By contrast, at later stages of infection, IAV becomes partially resistant to stress-induced translation arrest, thereby maintaining ongoing translation of viral gene products. To this end, the virus deploys multiple proteins that block stress-induced SG formation: (1) non-structural protein 1 (NS1) inactivates the antiviral double-stranded RNA (dsRNA)-activated kinase PKR, thereby preventing eIF2α phosphorylation and SG formation; (2) nucleoprotein (NP) inhibits SG formation without affecting eIF2α phosphorylation; (3) host-shutoff protein polymerase-acidic protein-X (PA-X) strongly inhibits SG formation concomitant with dramatic depletion of cytoplasmic poly(A) RNA and nuclear accumulation of poly(A)-binding protein. Recombinant viruses with disrupted PA-X host shutoff function fail to effectively inhibit stress-induced SG formation. The existence of three distinct mechanisms of IAV-mediated SG blockade reveals the magnitude of the threat of stress-induced translation arrest during viral replication.

Measles viruses deficient in C protein generate an increased amount of defective genomes that are bound by PKR, thereby causing eIF2α-mediated translational arrest, SG formation, and limiting viral gene expression. Christian Pfaller from the Cattaneo lab at the Mayo Clinic provided new insights into the structure and processing of viral PKR ligands. Measles virus infection results in the production of 5’-copyback defective-interfering RNA (DI-RNAs), which possess complementary 5’- and 3’termini. Because DI-RNAs can form dsRNA, they are efficiently bound by PKR and induce downstream signalling [39]. By sequencing DI-RNAs produced during WT and C-deficient measles virus infections, Christian observed frequent A-to-G or U-to-C hypermutation events with signatures consistent with ADAR1 modification sites.

The final talk of the symposium was delivered by Martijn Langereis from the van Kuppeveld lab, Utrecht University, who discussed the ways that picornaviruses evade stress pathway activation. Martijn constructed an infectious clone of encephalomyocarditis virus (EMCV) that was unable to prevent SG formation upon infection, and used it as a platform for screening a library of genes from different picornaviruses, including poliovirus, coxsackievirus, rhinovirus and foot-and-mouth disease virus (FMDV). He confirmed previous observations that viral proteinases from enteroviruses cleave essential SG constituent proteins. Additionally, he showed that the leader proteinase from aphthoviruses possessed similar cleavage activity. By contrast, cardio- and kobuviruses encode a small leader protein that inhibits SG formation by an as-yet-unidentified mechanism.

At this point, the symposium concluded with some final comments from Craig McCormick and the awarding of poster prizes to three deserving individuals, who had been selected by open ballot; PDF Gemma Perez Vilaro (Diez lab), Universitat Pompeu Fabra, who presented work on the regulation of p-body formation in HCV-infected livers; graduate student Anais Aulas (Vande Velde lab), Universite de Montreal, who presented work on the interaction between TDP-43 and G3BP1 in controlling SG function, and the relationship of these dynamics to motor neuron dysfunction in ALS; and Professor Vladimir Buchman, Cardiff University, who also presented work relating RNA granule dynamics to ALS, specifically describing the role of Fus/TLS fusion proteins in RNA granule aggregation.

Stress was resolved, and the group dispersed.
